# Network-guided genomic and metagenomic analysis of the faecal microbiota of the critically endangered kakapo

**DOI:** 10.1038/s41598-018-26484-4

**Published:** 2018-05-25

**Authors:** David W. Waite, Melissa Dsouza, Yuji Sekiguchi, Philip Hugenholtz, Michael W. Taylor

**Affiliations:** 1 School of Biological Sciences, University of Auckland, Auckland, New Zealand; 2 Australian Centre for Ecogenomics, School of Chemistry and Molecular Biosciences, The University of Queensland, Brisbane, Australia; 3 Department of Surgery, University of Chicago, Chicago, IL USA; 4 Marine Biological Laboratory, Woods Hole, MA USA; 5 Bio-Measurement Research Group, Biomedical Research Institute, National Institute of Advanced Industrial Science and Technology, Ibaraki, Japan; 6 Maurice Wilkins Centre for Molecular Biodiscovery, University of Auckland, Auckland, New Zealand

## Abstract

The kakapo is a critically endangered, herbivorous parrot endemic to New Zealand. The kakapo hindgut hosts a dense microbial community of low taxonomic diversity, typically dominated by *Escherichia fergusonii*, and has proven to be a remarkably stable ecosystem, displaying little variation in core membership over years of study. To elucidate mechanisms underlying this robustness, we performed 16S rRNA gene-based co-occurrence network analysis to identify potential interactions between *E. fergusonii* and the wider bacterial community. Genomic and metagenomic sequencing were employed to facilitate interpretation of potential interactions observed in the network. *E. fergusonii* maintained very few correlations with other members of the microbiota, and isolates possessed genes for the generation of energy from a wide range of carbohydrate sources, including plant fibres such as cellulose. We surmise that this dominant microorganism is abundant not due to ecological interaction with other members of the microbiota, but its ability to metabolise a wide range of nutrients in the gut. This research represents the first concerted effort to understand the functional roles of the kakapo microbiota, and leverages metagenomic data to contextualise co-occurrence patterns. By combining these two techniques we provide a means for studying the diversity-stability hypothesis in the context of bacterial ecosystems.

## Introduction

The kakapo (*Strigops habroptilus*) is an endemic New Zealand parrot, known for its unusual diet, nocturnal behaviour and lack of flight. The kakapo is classified as ‘critically endangered’ with a current population of approximately 150 individuals confined to predator-free islands off the coast of New Zealand. The diet of the kakapo is something of a peculiarity – although kakapo are nominally herbivorous they do not ingest large volumes of plant material, instead preferring to crush plant fibres in their beaks, extracting the juices and discarding the remainder^1,2^. The diet of the kakapo generally revolves around shoots and leaves, with the exception of the infrequent breeding seasons during which kakapo feed extensively on the fruit of the native rimu (*Dacrydium cupressinum*). Breeding in kakapo is entrained to the mast fruiting of this tree, possibly owing to its high concentration of calcium and vitamin D^3^, but even during this period of low-fibre feeding cellulose is the most abundant carbohydrate in the kakapo crop^4^.

Gastrointestinal (GI) tract-associated bacteria have been of interest for over half a century^5^ and have been linked to improved energy harvest from food sources^6–8^, vitamin and nutrient synthesis^9–11^, and gut development^12,13^. Diet is a powerful factor in shaping the gut microbiota^14–16^ as it governs the nutrients available to that community. Even when the host feeds on a single food source a diverse range of carbohydrates, fats, and proteins are introduced to the gut. The resident microbiota feed on these nutrients with individual species, or interacting webs of species, utilising different nutrients from the ingesta^17^. In vertebrates with and without anatomical adaptation to herbivory (e.g. ruminants and panda, respectively) the resident microbiota plays a critical role in the depolymerisation of cellulose and hemicellulose into sugars that are accessible to the host^8,18,19^.

Microbial degradation of cellulose is likely to occur in the kakapo as well, but the functional role of kakapo-associated microbes currently remains unexplored. With the exception of two studies of specific pathogens^20,21^, the kakapo microbiota has only recently been investigated, with analysis of bacterial 16S rRNA gene sequences revealing low-diversity, but distinct, bacterial communities in the crop (foregut) and hindgut^22^. Unlike most vertebrate gut communities, the kakapo microbiota is dominated by *Gammaproteobacteria* and Firmicutes, with Actinobacteria and Bacteroidetes observed sporadically, and at low abundances in most individuals^23–25^. Interestingly, the gut microbiota is typically dominated by a single bacterial species, identified through cultivation as *Escherichia fergusonii*^26^. Monitoring over a number of years has revealed that the kakapo microbiota is surprisingly robust^23,25^, although the mechanism(s) underlying such stability and how members of the microbiota interact within the gut remain unexamined.

Metagenomic analysis of microbial data sets has allowed for unparalleled insights into the functional ability of the gut microbiota and represents a powerful tool for discovering the role of the microbiota within an animal’s gut. Metagenomic data are frequently interpreted as though all bacteria are capable of interacting; however, factors such as spatial separation, physicochemical characteristics and even community turnover create barriers and local niches that can limit the ability of one bacterium to interact with another. Confirming suspected interactions requires intense, often highly selective, study that negates the benefits offered by high-throughput techniques. Co-occurrence networks of microbial communities represent a means to bridge such limitations, allowing researchers to identify population subsets within a microbiota that may be interacting in either a beneficial or antagonistic manner. In this approach, individual species (or operational taxonomic units – OTUs) are represented by nodes in a web, connected by predicted interactions with other species^27–29^. This allows the development of more specific research questions as well as an objective basis for partitioning large data sets into biologically relevant subsets, and provides a means to identify keystone species^30^. Network analysis also provides other metrics to analyse the community through measures such as the degree of community fragmentation^31^.

The relationship between community diversity and stability is a long-studied issue within ecology^32,33^. At its core, the hypothesis states that a more diverse community of co-occurring organisms is more likely able to compensate for environmental fluctuations, and maintain its functional capacity over long periods^32^. While diversity may therefore be considered a proxy for stability, it is not necessarily the causal agent and may instead result as a consequence of the ecological forces which underpin stability^34–36^. The kakapo gut microbiota often appears to be a near monoculture of *Escherichia*, yet this community is undoubtedly resilient to both variation through time and anthropogenic interference^23,25^. While *Escherichia*-like sequences are frequently observed in the microbiota of other birds, including parrots^37–43^, the uneven distribution of microbes in the kakapo hindgut appears unique. In order to elucidate mechanisms underlying this observation, we performed co-occurrence network analysis on 16S rRNA gene amplicon data in an attempt to characterise potential ecological interactions of *Escherichia* within the gut microbiota. We further employed genomic sequencing of cultured representatives of dominant members of the microbiota and shotgun metagenome sequencing of the uncultivated microbiome to identify a functional basis for the findings of the network analysis.

## Materials and Methods

### Amplicon sequencing and analysis

Fresh faecal samples were obtained from three adult and three juvenile kakapo and DNA was extracted using a previously reported bead-beating technique^22^. PCR was performed using the 533 F (5′-GTG CCA GCA GCY GCG GTM A-3′) and 907 R (5′-CCG TCA ATT MMY TTG AGT TT-3′) Bacteria-targeting primers, to amplify an approximately 350 bp region of the 16S rRNA gene sequence. Cycling conditions consisted of an initial denaturing step at 94 °C for 5 min, followed by 20 touchdown cycles of 94 °C for 30 s, 60 °C for 30 s, and 72 °C for 45 s with a 0.5 °C decrease in annealing temperature per cycle. Touchdown was followed by 10 additional cycles of 94 °C for 30 s, 50 °C for 30 s, and 72 °C for 45 s followed by a final elongation step at 72 °C for 10 min^23^. PCR products were purified using the Agencourt AMPure XP kit (Beckman Coulter Life Sciences, Brea CA, United States) and amplicon size examined using an Agilent 2100 Bioanalyzer. DNA concentration was quantified using the Quant-iT PicoGreen dsDNA assay kit (Thermo Fisher Scientific, Waltham MA, USA) according to manufacturer instructions. DNA was pooled at equimolar concentrations and pyrosequencing was performed by Macrogen Inc (Seoul, South Korea) using the GS-FLX Titanium platform.

A subset of 16S rRNA gene amplicon data from three time points of a previous study of the gut microbiota of 10 juvenile and 10 adult kakapo were utilized in this study^23^ (Table S1). Samples from the captivity cohort of the original study were removed from the data set to remove the confounding effect of antibiotic treatment on community dynamics. This selection criterion resulted in a total of 36 amplicon samples from previous data and six additional amplicon samples amplified from the samples used for metagenome sequencing. Raw data were processed using mothur (version 1.36.1)^44^ following the standard operating procedure for pyrosequencing data. Flowgrams were trimmed to equal length and denoised, then the resulting sequences were trimmed of barcodes and primer sequences. Trimmed sequences were then aligned to the SILVA reference alignment (version 119) and short sequences, together with those containing ambiguous base calls or homopolymer runs greater than 8 nucleotides, removed. The aligned sequences were then end-trimmed and chimeric sequences identified using UCHIME^45^ were removed from the data set. The remaining high-quality sequences were classified against an alignment-trimmed SILVA small subunit database (version 119)^46,47^ using the naïve Bayesian method^48^. Sequences identified as archaeal, eukaryotic, chloroplast, or mitochondria were removed, as were sequences that could not be classified to at least phylum level. Operational taxonomic units (OTUs) were clustered at 99% sequence identity from the remaining sequences and the taxonomic affiliation of each OTU taken as the consensus taxonomy of the individual sequences contributing to that cluster. OTUs present in fewer than five samples were removed from the OTU table, which was then randomly subsampled to a depth of 2,000 sequences per sample. The community structure of the OTU data set was visualised using non-metric multidimensional scaling of a Bray-Curtis distance matrix calculated from the OTU table using the vegan package in R^49,50^.

### Amplicon network analysis

Correlation scores between pairs of OTUs were calculated in SparCC using 20 refining iterations, after which statistical significance was assigned to each correlation using a pseudo-p value approximation with 1,000 permutations^51^ then analysed in the R software environment using the igraph package^49,52^. OTU correlations were encoded as a graph whereby OTUs (nodes) were joined via unweighted edges if their correlation coefficient was greater than or equal to 0.3 and statistically significant following a Benjamini-Hochberg correction for multiple testing (FDR ≤ 0.05). Although some analyses have constructed a unified graph of all interactions^53–55^, we opted to restrict our data to positive correlations only. This approach was chosen as the ecological interpretation and consequence of positive/negative correlations differ, and the commonly utilised network statistics do not account for polarity of an interaction. The network statistic transitivity (also known as clustering coefficient) was calculated for the complete network and interpreted in the context of a clustering ratio, comparing the clustering density of the kakapo graph to randomly constructed graphs with the same number of nodes and edges. As the topology of graphs, and their network statistics, can vary greatly with random permutation (Fig. S1), 1,000 random graphs were constructed to create a null distribution of transitivity scores, and the median value used to calculate the clustering ratio of the kakapo network. Node-specific statistics degree and betweenness were calculated for each OTU using the methods and definitions described by Williams *et al*.^53^. The resulting network was visualised using Cytoscape^56^ and figures prepared for publication using Inkscape.

### Extraction and sequencing of genomic and metagenomic DNA

Eight bacterial isolates, previously obtained from kakapo faeces (Table S2)^26^, were grown to stationary phase and genomic DNA extracted using a standard enzymatic digest protocol with minor modifications^57^. Cells were suspended in TE buffer and 740 µL of suspension transferred to a fresh 1.5 mL microcentrifuge tube. Forty microlitres of lysozyme (100 mg/mL; Sigma-Aldrich, St Louis MO, USA), 40 µL 10% SDS and 16 µL proteinase K (10 mg/mL; Sigma-Aldrich, St Louis MO, USA) were added, and tubes were incubated overnight at 37 °C with gentle mixing. Following cell lysis, 100 µL NaCl (5 M) and 100 µL CTAB/NaCl mixture (described in original protocol) were added and samples further incubated at 65 °C for 10 min. Five hundred microlitres of chloroform:isoamyl alcohol (24:1) was added to each sample and tubes were mixed by inversion then centrifuged for 15 min at 13,000 rpm. One millilitre of supernatant was transferred to a fresh tube and the process repeated with 500 µL phenol:chloroform:isoamyl alcohol (25:24:1). Supernatant (~1 mL) was transferred to a fresh 2 mL microcentrifuge tube to which 0.6 vol isopropanol and 0.1 vol sodium acetate (3 M, pH 5.2) were added. Samples were mixed by inversion and incubated at room temperature for 1 h then centrifuged at 16,000 g at 4 °C for 30 min. The supernatant was removed and the resulting pellet washed twice with ice-cold 70% ethanol. Samples were air dried then resuspended in 20 µL TE buffer with RNase A (Qiagen, Germantown, MS, USA), incubated for 20 min at 37 °C, then finally stored at −20 °C. DNA concentration was calculated using a QuBit Quant-iT DNA high-sensitivity assay and DNA was electrophoresed on a 2% agarose gel to assess shearing.

For metagenomic analysis, faeces were fractionated by suspending one gram of faecal material in 5 mL PBS and vortexing for 2 min then centrifuging at 800 g for 2 min^58^. The upper fraction was collected and centrifuged at 7,500 g for 7 min, then supernatant removed and pelleted biomass washed twice with 1 mL PBS. DNA extraction was performed as above with a single modification: following addition of CTAB/NaCl, samples were incubated at 94 °C for 30 min, then briefly cooled on ice. For both genomic and metagenomic samples, library preparation and sequencing were performed by New Zealand Genomics Ltd. Raw DNA was prepared using the Nextera XT kit and samples pooled for sequencing. All 14 samples were sequenced together in three separate sequencing runs, to allow for adjustment of template ratios between runs to correct over-/under-represented samples. Sequencing was performed using the Illumina MiSeq with 2 × 250 bp paired-end reads.

### Genomic and metagenomic analysis

Raw genomic reads were filtered and assembled using the PAGIT toolkit^59^. Briefly, reads were quality trimmed using sickle^60^ in paired-end mode, with a quality threshold of 20 and minimum read length of 50 bp. Genomes were then assembled de novo using velvet^61^, with kmer sizes determined manually for each genome. Gap closing was then performed using IMAGE^62^, and error correction with ICORN^63^ with default settings. Metagenomic sequences from all samples were processed using the same workflow, but pooled for assembly in MetaVelvet^64^. Gene prediction for both genomes and the metagenome was performed using Prodigal^65^ and predicted protein sequences annotated using BLAST against the NCBI non-redundant protein database requiring a minimum sequence identity ≥50%, and an e-value of ≤ 1e-5. For genomic comparison, we downloaded the genomes of 177 *Enterobacteriaceae* type species, including *Escherichia coli*, *E. fergusonii*, and *E. albertii*, from the NCBI genome database and annotated these using the same approach. Functional pathway analysis was performed using MEGAN^66^. Isolate genomes were further annotated against the CAZy database using the dbCAN web server^67,68^. For phylogenetic inference of the beta-1,4-endoglucan hydrolase orthologues, protein sequences were aligned using MAFFT with the high-sensitivity (L-INS-i) algorithm^69,70^. The alignment was then trimmed with TrimAl^71^ and phylogenetic inference performed with RAxML^72^ under the Le and Gascuel model of amino acid substitution^73^ and 100 bootstrap resamplings to assess node support.

Per-sample gene abundances were calculated by mapping the quality filtered, unassembled sequences from each sample to the assembled contigs using bowtie2^74^. Reads mapped to each contig were then normalised by length to account for longer contigs receiving a larger proportion of reads mapping. Annotated genes within the metagenome were separated based on the taxonomic origin of sequences, using the least-common ancestor method in MEGAN to identify and extract bins of metagenomic sequence data. All novel sequence data were submitted to the NCBI Sequence Read Archive under accession number PRJNA381379.

## Results and Discussion

**Figure 1:**
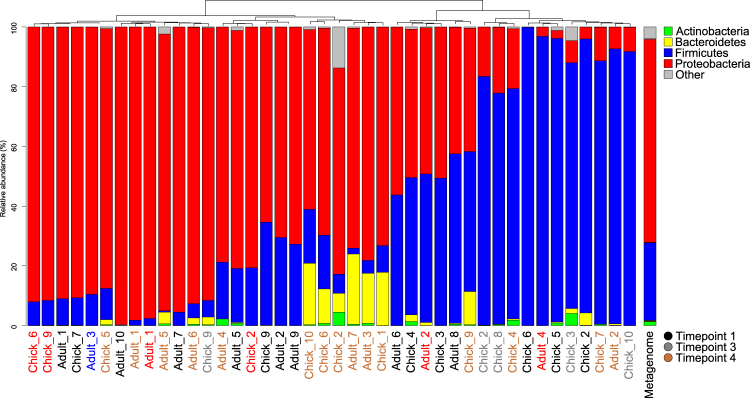
Phylum-level distribution of sequences in the amplicon data. Taxonomic abundance data were summarised at the phylum level and clustered using furthest-neighbour hierarchical clustering. Clustering was performed using the full phylum-level profile, with the following phyla aggregated to ‘Other’ for ease of viewing: Acidobacteria, Armatimonadetes, Chloroflexi, Fusobacteria, Gemmatimonadetes, Lentisphaerae, Planctomycetes, Saccharibacteria, Spirochaetes, Verrucomicrobia, WPS-1, WPS-2, and unclassified. Time points refer to sampling strategy in Table S1. Samples selected for metagenome sequencing and amplified with amplicon sequence are marked in red. ‘Metagenome’ column refers to the taxonomic overview of the assembled and annotated metagenome.

To ensure that samples selected for metagenome sequencing were representative of the kakapo microbiome, we compared 16S rRNA gene amplicon data from each candidate metagenome sample to previously sequenced kakapo amplicon data. No strong influence of environmental or age-related factors was observed in the amplicon data at either the taxonomic or OTU level (Fig. 1, Fig. S2), and samples intended for metagenome sequencing represented the Proteobacteria-rich, Firmicutes-rich, and intermediate community states (Fig. 1). With no clear environmental or age-related factors separating the microbiota of kakapo samples, and hence biasing findings, we examined the data set for evidence of interactions between bacteria in the microbiota. Rarefaction analysis was performed to verify that subsampling the OTU table to 2,000 sequences per sample provided sufficiently saturated counts (Fig. S3). OTUs observed in fewer than five samples were removed from the data set, resulting in a median Jaccard similarity of 0.27 between samples, consistent with the recommendations of Berry and Widder^30^. Correlations between pairs of OTUs were calculated using SparCC, and a network graph constructed from OTUs (nodes) if their correlations (edges) were ≥0.3 and statistically significant (FDR ≤ 0.05). The degree of clustering in the resulting network was compared to a null distribution of randomly constructed networks of equal numbers of nodes and edges. The cluster ratio of the kakapo network was 4.6, indicating approximately four-fold greater clustering than could be expected by chance in an equally dense network. This finding is biologically relevant as it indicates that bacterial OTUs within the microbiota are forming small, highly interacting sub-populations rather than existing in a more generalised system (for example, Fig. S1). Overall, this suggests that rather than a single metacommunity, the kakapo microbiota acts as a set of smaller components that are nominally independent of each other.

**Figure 2:**
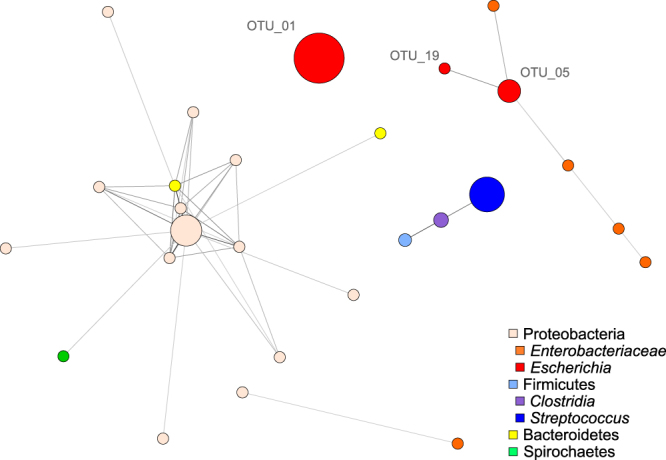
Interaction networks of the kakapo microbiota. 16S rRNA gene-based correlation network of the kakapo microbiota, displaying statistically significant interactions with a correlation coefficient of ≥0.3. Node size is scaled based on the overall abundance of each OTU in the microbiota. ‘*Enterobacteriaceae*’ nodes represent OTUs that could not be classified beyond the family level, and does not include sequences classified as *Escherichia*. OTU labels of *Escherichia* nodes refer to OTU identifiers in Table 1.

Previous research into the kakapo microbiota has identified two bacterial species, *Escherichia fergusonii* and *Streptococcus gallolyticus*, as present in all kakapo samples^22,23,26^, although more recent analysis indicates that the core kakapo microbiome consists of only *E. fergusonii*^25^. Regardless of the exact membership of the core microbiota, we were interested in identifying microbes that may be involved in mutualistic interactions with these two organisms. Defining OTUs at 99% sequence identity yielded three OTUs belonging to the gammaproteobacterial genus *Escherichia* (*Escherichia*-OTUs). The most abundant of these OTUs (29.4% of reads across all samples) was not associated with any other OTUs in the network (Fig. 2). Two additional *Escherichia*-OTUs (totalling 10.0% of reads) were correlated with each other and OTUs belonging to the *Gammaproteobacteria* which could not be classified below family level (*Enterobacteriaceae*-OTUs). Consistent with previous research a single *Streptococcus*-OTU (20.3% of reads) was detected and correlated with an OTU belonging to the genus *Clostridium* (Fig. 2).

Network analysis provides several metrics which can be used to indicate the importance of particular species to the wider community^30,75^ and owing to their frequent occurrence in the kakapo microbiota we aimed to ascertain the ecological importance of the *Escherichia*- and *Streptococcus*-OTUs in the microbiota. We scored all OTUs using the degree (number of connecting edges, normalised to graph size) and betweenness (number of shortest connecting paths that travel through an OTU) statistics then ranked each OTU by score. Consistent with their low incidence of co-occurrence in the visualised graph, *Escherichia*-OTUs generally did not score highly under either metric (Table 1). The apparent lack of correlations between these core OTUs may suggest that Escherichia-OTUs thrive in the gut due to their metabolic capacity that affords them the luxury of non-reliance on syntrophic interactions with other gut bacteria.

**Table 1:** Rank of the OTUs reported according to commonly reported keystone metrics.

OTU Label	Classification	Normalised Degree	Betweenness
OTU_01	*Escherichia*	—	—
OTU_02	*Streptococcus*	12	8
OTU_03	Proteobacteria	1	1
OTU_05	*Escherichia*	6	3
OTU_06	*Clostridium*	11	5
OTU_08	*Turicibacter*	13	9
OTU_09	*Arenicella*	3	6
OTU_13	*Enterobacteriaceae*	14	10
OTU_15	*Alphaproteobacteria*	4	7
OTU_19	*Escherichia*	15	11
OTU_20	*Rhodobacteraceae*	5	4
OTU_23	*Enterobacteriaceae*	16	12
OTU_24	*Enterobacteriaceae*	17	13
OTU_25	*Spirochaeta*	18	14
OTU_26	*Enterobacteriaceae*	19	15
OTU_28	*Enterobacteriaceae*	20	16
OTU_31	*Alphaproteobacteria*	21	17
OTU_37	*Alphaproteobacteria*	9	18
OTU_48	*Flavobacteriaceae*	2	2
OTU_49	*Rhodobacteraceae*	7	19
OTU_50	*Pseudomonas*	22	20
OTU_64	*Roseobacter*	23	21
OTU_69	*Rhodobacteraceae*	24	22
OTU_88	*Alphaproteobacteria*	10	23
OTU_153	*Ekhidna*	25	24
OTU_245	*Alphaproteobacteria*	8	25
OTU_393	*Gammaproteobacteria*	26	26

OTUs are ranked according to their normalised degree and unweighted betweenness scores. A total of 20 nodes were maintained in the positive graph once low scoring correlations were removed. OTUs identified as *Escherichia* and *Streptococcus* are highlighted. Note that scores could not be calculated for OTU_01 due to its lack of edges in the network graph.

The kakapo gut microbiota is stable through time and resilient to changes in diet and anthropogenic perturbations, yet is one of the lowest diversity avian microbiotas known^76^. Bacteria of the genus *Escherichia* are often detected in the guts of avians^37–43^ but they have not been reported at the abundances found in the kakapo. A long-standing hypothesis to explain the ubiquity of *Escherichia* in the gut of kakapo was that it was a consequence of supplemental feeding and human intervention. However, a recent analysis of kakapo which have never received supplemental feeding does not support this hypothesis^25^. While it is important to recognise that the lack of observed correlation does not exclude the possibility of ecological interactions between *Escherichia*-OTUs and other microbes in the kakapo gut, this finding does provide evidence that such an interaction would not be a strong effect. In order to contextualise these observations we sequenced the genomes of six *E. fergusonii* and two *S. gallolyticus* isolates cultivated from the kakapo gut. We further supplemented these data with shotgun metagenomic sequencing to obtain functional data for bacteria which have previously evaded culture.

Quality filtering of genomic and metagenomic sequences resulted in between 1.3 and 2.9 million reads per bacterial isolate, and approximately 17.9 million paired reads from metagenome samples (Table S3). Assembly of isolate genomes yielded draft genomes with sizes and predicted gene counts appropriate for their respective organisms (Table S3). Gene prediction and annotation of the kakapo metagenome yielded a total of 257,679 predicted genes, of which 90.3% (233,154) were bacterial. Only 14 genes of archaeal origin were detected, consistent with our previous finding that archaea are extremely rare or absent from the kakapo gut^22^. The remaining genes were of eukaryotic (avian and fungal, 0.9%) or viral (0.03%) origin, or were unable to be annotated (8.6%). The taxonomic profile of the bacterial metagenome was consistent with the results of amplicon sequencing (Fig. 1), dominated by genomic material belonging to Proteobacteria and Firmicutes, with a smaller number of Actinobacteria and Bacteroidetes genes detected. The majority of sequences were identified as belonging to the KEGG pathways associated with carbohydrate metabolism and amino acid turnover (Table 2).

**Table 2:** Overview of functional profile of metagenomes.

KEGG Category	Adult_1	Adult_2	Adult_4	Chick_6	Chick_7	Chick_9
Cellular Processes	3.91	7.54	4.00	2.88	6.67	5.01
Environmental Information Processing	19.66	19.48	15.54	15.28	16.77	19.61
Genetic Information Processing	14.30	12.91	21.32	18.05	15.15	13.52
Human Diseases	2.86	2.98	1.87	2.25	2.85	2.68
Metabolism	55.68	53.41	56.30	57.44	54.57	55.01
*Amino Acid Metabolism*	*11.79*	*11.83*	*11.05*	*11.19*	*10.71*	*12.45*
*Biosynthesis of Other Secondary Metabolites*	*1.08*	*0.83*	*0.89*	*1.00*	*0.78*	*0.87*
*Carbohydrate Metabolism*	*14.41*	*13.63*	*15.42*	*15.2*	*13.83*	*14.41*
*Energy Metabolism*	*7.82*	*7.17*	*5.87*	*8.43*	*8.09*	*7.03*
*Glycan Biosynthesis and Metabolism*	*2.15*	*1.59*	*2.50*	*1.88*	*2.06*	*1.94*
*Lipid Metabolism*	*3.23*	*3.44*	*3.35*	*3.03*	*3.59*	*3.85*
*Metabolism of Cofactors and Vitamins*	*5.15*	*4.79*	*5.04*	*5.21*	*4.66*	*4.47*
*Metabolism of Terpenoids and Polyketides*	*1.01*	*1.31*	*1.58*	*1.69*	*1.48*	*1.40*
*Nucleotide Metabolism*	*6.62*	*6.34*	*8.79*	*7.69*	*7.56*	*5.91*
Xenobiotics Biodegradation and Metabolism	2.41	2.5	1.81	2.13	1.79	2.67
Organismal Systems	2.11	2.13	1.09	2.43	2.75	2.11
Unclassified	1.48	1.56	1.44	1.68	1.25	2.07

Columns denote the relative abundance (%) of each functional category the overall metagenome for sequences of bacterial origin. Italicised categories are a subcategory of the main entry ‘Metabolism’ and sum to the total abundance of ‘Metabolism’.

The kakapo diet is low in starch^2,4,77^ and the most abundant carbohydrate sources in the diet of kakapo are cellulose and other fibrous material. Depolymerisation of plant fibres such as cellulose, hemicellulose and xylan is a difficult biological process and is frequently outsourced to the gut microbiota^19,78,79^. Cellulose degradation typically occurs in a two-step method whereby cellulose polymers are fragmented into shorter cellobiose oligomers, which are in turn hydrolysed to glucose by cellobiases. The initial step of depolymerising cellulose to cellobiose in the kakapo microbiome was attributed exclusively to members of the family *Enterobacteriaceae* (including *Escherichia*) and was observed in all *E. fergusonii* genomes. Amongst the isolate genomes, however, the abundance of glucoside hydrolases was not sufficiently high (0.96–1.16% of genome) to consider these isolates cellulolytic specialists^80^. Genes responsible for the subsequent liberation of glucose from cellobiose were present in many other bacterial lineages including the genera *Streptococcus* and *Clostridium* (Table 3). It appears that while these lineages are capable of metabolising cellulose by-products, they are incapable of digesting long-chain cellulose. In a similar vein, we observed genes coding for the utilisation of xylose within the *Escherichia* genomes and metagenome, but no apparent ability to perform the initial depolymerisation of xylan. Genes responsible for the depolymerisation of xylan were present in the metagenome, belonging exclusively to non-*Escherichia* members of the *Enterobacteriaceae* (Table 3), providing a potential mechanism explaining the correlation between *Escherichia*- and *Enterobacteriaceae*-OTUs (Fig. 2). Genes related to the utilisation of other plant polysaccharides such as sucrose and maltose were observed in the *E. fergusonii* genomes and the *Enterobacteriaceae*-, *Streptococcus*-, and *Clostridium*-attributed proteins of the metagenome. Amylases were observed in the *E. fergusonii* and *S. gallolyticus* genomes, as well as all four groups of interest in the metagenome.

**Table 3:** Key carbohydrate utilisation enzymes of bacteria in the kakapo gut.

EC accession	Substrate	Product	*Enterobacteriaceae* (metagenome)	*Escherichia* (metagenome)	*Escherichia* (genome)	*Streptococcus* (genome and metagenome)	*Clostridium* (metagenome)
3.2.1.4	Cellulose	Cellobiose	*	*	*		
3.2.1.21	Cellobiose	Glucose	*	*	*	*	*
3.2.1.37	Xylan	Xylose	*				
5.3.1.5	Xylose	Xylulose	*	*	*	*	*
2.7.1.17	Xylulose	Xylulose-5P	*	*	*	*	*
5.1.3.4	Xylulose-5P	Ribulose-5P	*	*	*	*	*

Differentiating pathways for carbohydrate utilisation. From the end points of glucose and ribulose-5P, energy is generated through glycolysis and the pentose phosphate pathway, respectively.

*E. fergusonii*, or OTUs suspected to represent this species, are frequently the most abundant lineage found in the kakapo gut and the species has retained this status over six years of molecular surveying^22,25^. The kakapo microbiota is remarkably stable and even after antibiotic stress is able to recover to the ‘normal’ state within weeks^23^. Our data represent the first metagenomic insights into the kakapo microbiome and provide a hypothesis to explain the mechanism by which *E. fergusonii* is maintained despite its apparent weak interaction with the remainder of the microbiota. Amongst the microbial lineages investigated, *E. fergusonii* alone has the genomic potential to utilise all forms of carbohydrate encountered by the kakapo, independent of mutualistic interactions with other members of the microbiota. For example, when the kakapo is not receiving supplemental feeding *E. fergusonii* appears able to metabolise cellulose but if the kakapo gains access to supplemental feed, *E. fergusonii* possesses the genomic capacity to utilize the starch that this food source provides. By contrast, other members of the microbiota are only capable of using a subset of the available resources and will presumably go through periods of starvation during which they will be unable to reproduce. Analysis of the genomic content of isolate genomes did not reveal significant difference between kakapo isolates and the previously cultivated *Enterobacteriaceae* type material (Fig. S4). The low abundance of glucoside hydrolase enzymes in the genomes, and the standard vertical inheritance of beta-1,4-endoglucan hydrolase (Fig. S5), further indicate that the *E. fergusonii* genomes have not evolved novel celluloytic functionality to colonize the kakapo gut. Some *Escherichia*-OTUs were predicted to interact with *Enterobacteriaceae*-OTUs, which we hypothesise possess the ability to depolymerise xylan based on metagenomic data. If correct, this interaction provides an additional avenue for the metabolism of *E. fergusonii* via xylose by-products.

Based on its predicted ability to metabolise a wide range of plant sugars, *E. fergusonii* appears to be extremely well suited to the kakapo gut as a metabolic generalist, able to utilise both natural and supplemental energy sources. In contrast, the other microbes such as *S. gallolyticus* appear able to make use of only a fraction of the available carbohydrate sources. Members of the Firmicutes such as *Clostridium* and *Streptococcus* may be able to make use of partially digested plant fibre produced by *E. fergusonii* or other *Enterobacteriaceae*, although our data suggest that this would not be a consistent occurrence. Even when discounting the numerical abundance of OTUs and presence/absence measures are considered, the kakapo microbiota is stable through time despite its low phylogenetic diversity^24,25^. We attribute this apparent stability to the considerable diversity of carbohydrate metabolism pathways within the kakapo microbiota, primarily attributed to *E. fergusonii* and the limited diet of the kakapo. These data demonstrate that taxonomic diversity alone does not accurately reflect the ‘true’ functional diversity within an ecosystem, and that the diversity-stability interaction should not be thought of solely in terms of taxonomic diversity.

It is possible that the ubiquity of *E. fergusonii* in the kakapo microbiota is the result of an opportunistic colonization event, where it has supplanted the original kakapo microbiota, or that this bacterium has only risen to prominence in response to the supplemental feeding practice. Such an event would be consistent with the lack of differentiation in isolate genomes that would suggest adaptation to the kakapo hindgut. We do not consider this scenario likely, however, as the microbiota from kakapo that have never received supplemental feeding are also rich in *E. fergusonii*-like OTUs^25^. Furthermore, such a hypothesis would stand in strict opposition to the observation that microbiomes tend to evolve through time with their host lineage.

We have performed an in-depth examination of the kakapo gut microbiota with the aim of understanding the ecological interactions among key gut microbes. Insights into potential mutualisms occurring within this habitat were gained using correlation network analysis, then further explored through genome and metagenome sequencing. Our findings suggest that E. fergusonii strains have the metabolic capacity to persist and grow in the kakapo gut. Futhermore, the lack of a robust and reproducible correlations with other members of the gut microbiome suggests that this growth occurs without reliance upon syntrophic partners. We attribute this observation to functional flexibility within the *Escherichia* genome. These data are important not only for their relevance to understanding the kakapo microbiota, but they provide evidence that taxonomic diversity is not the only means through which ecological stability can be achieved. This finding is an important consideration for understanding microbial environments which are known for, and often discussed in terms of, their phylogenetic diversity. More specifically, our data provide a basis for beginning to understand the forces which govern the kakapo microbiome and will provide a framework for future investigation.

## Electronic supplementary material

Supplemental Information

Supplemental OTU Table
