# Supplementary material for: Network-guided genomic and metagenomic analysis of the faecal microbiota of the critically endangered kakapo
Source: Sci Rep. 2018 May 25;8:8128. doi: 10.1038/s41598-018-26484-4 (PMC5970201; doi:10.1038/s41598-018-26484-4)
Supplement: Supplementary file 1 — Supplemental Information [file 41598_2018_26484_MOESM1_ESM.docx]

# Network-guided genomic and metagenomic analysis of the faecal microbiota of the critically endangered kakapo

David W. Waite^1,2^*, Melissa Dsouza^3,4^, Yuji Sekiguchi^5^, Philip Hugenholtz^2^, Michael W. Taylor^1,6^

^1^School of Biological Sciences, University of Auckland, Auckland, New Zealand

^2^Australian Centre for Ecogenomics, School of Chemistry and Molecular Biosciences, The University of Queensland, Brisbane, Australia

^3^Department of Surgery, University of Chicago, Chicago, IL, USA

^4^Marine Biological Laboratory, Woods Hole, MA, USA

^5^Bio-Measurement Research Group, Biomedical Research Institute, National Institute of Advanced Industrial Science and Technology, Ibaraki, Japan

^6^Maurice Wilkins Centre for Molecular Biodiscovery, University of Auckland, Auckland, New Zealand

*Corresponding Author

David W. Waite

School of Chemistry and Molecular Biosciences

University of Queensland

d.waite@uq.edu.au

##
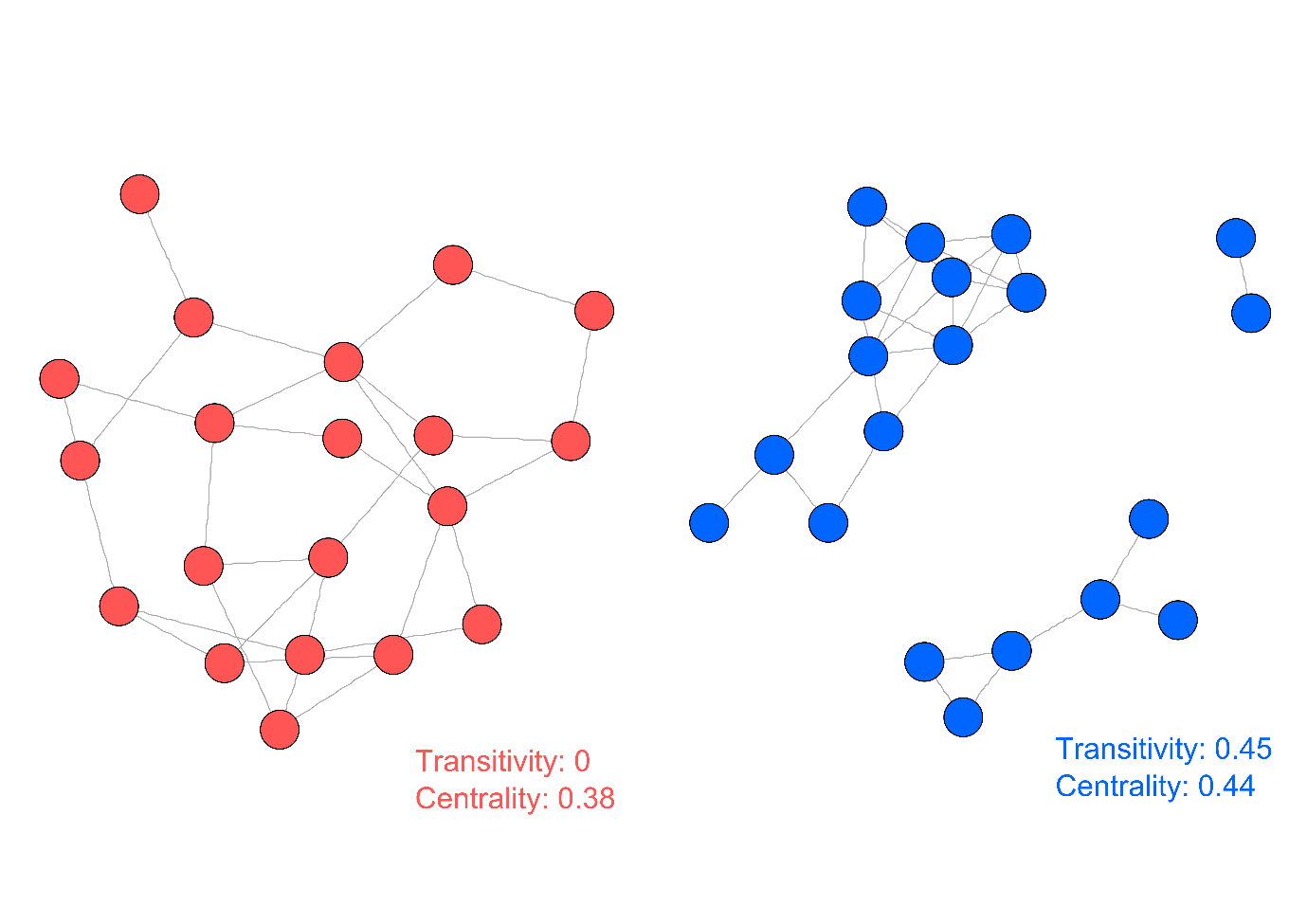


**Supplemental Figure S1. Comparison of networks displaying low and high modularity**

Randomly constructed graphs of 20 nodes and 30 edges (connections) were constructed in R using the *erdos.renyi.game* function of the igraph package. 10,000 graphs were randomly constructed and those with the lowest (red) and highest (blue) transitivity value were retained. Both graphs are constructed from the same number of nodes and connections, but stochastic placement of the connections drastically alters the topology of the network and associated statistics. Such differences in topology have ecological significance in the interpretation of the interactions represented within the network.


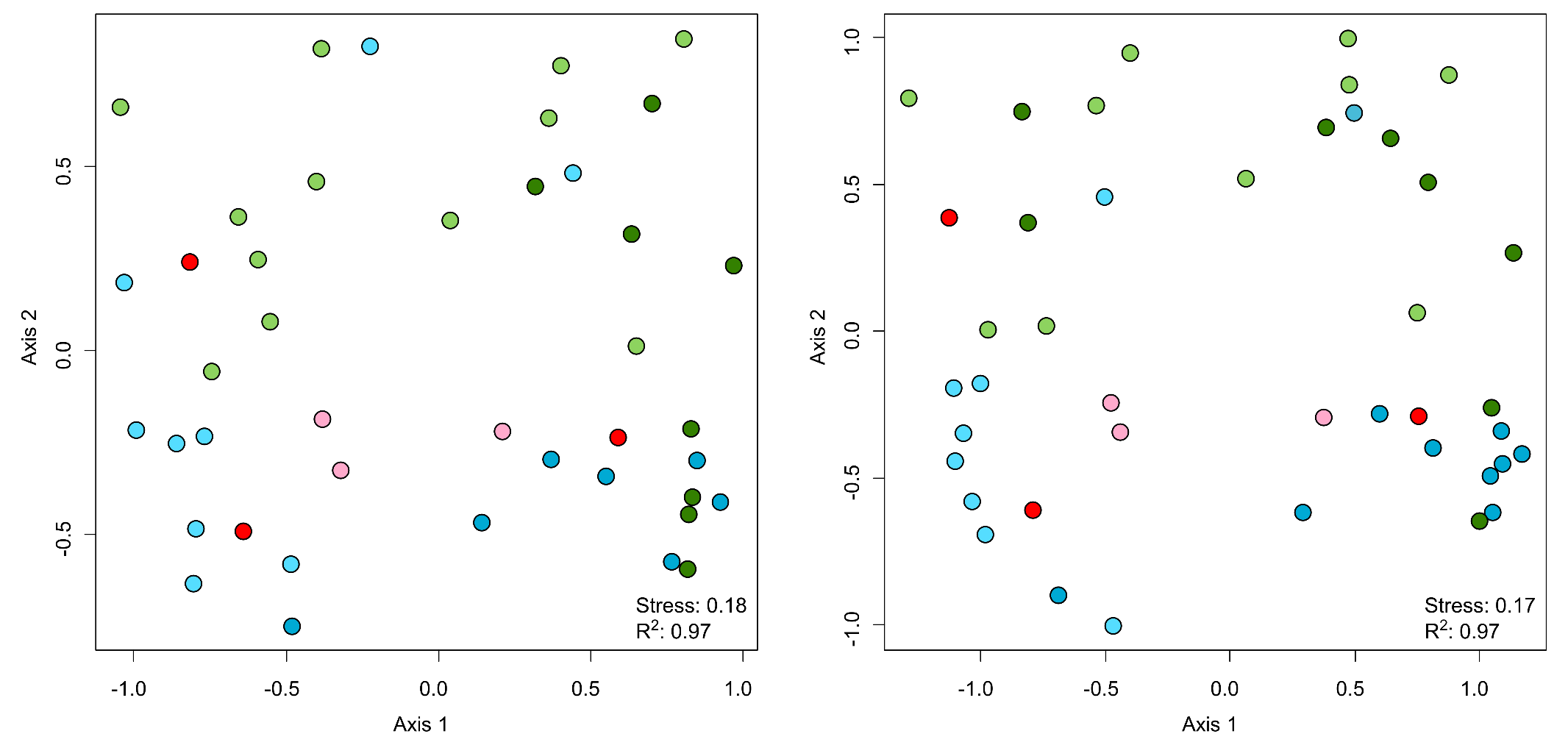


**Supplemental Figure S2. Non-metric multidimensional scaling plot of the kakapo 16S rRNA gene OTU data**

Ordination was performed using the Bray-Curtis distance with the full data set (left) and as subsampled for network analysis (right). Samples from kakapo chicks (green) and adults (blue) are coloured by year sampled (pale = 2011, dark = 2012). Samples used for metagenomic sequencing are marked in pink (chick) and red (adult), and show no consistent difference from other samples used in network construction.

**
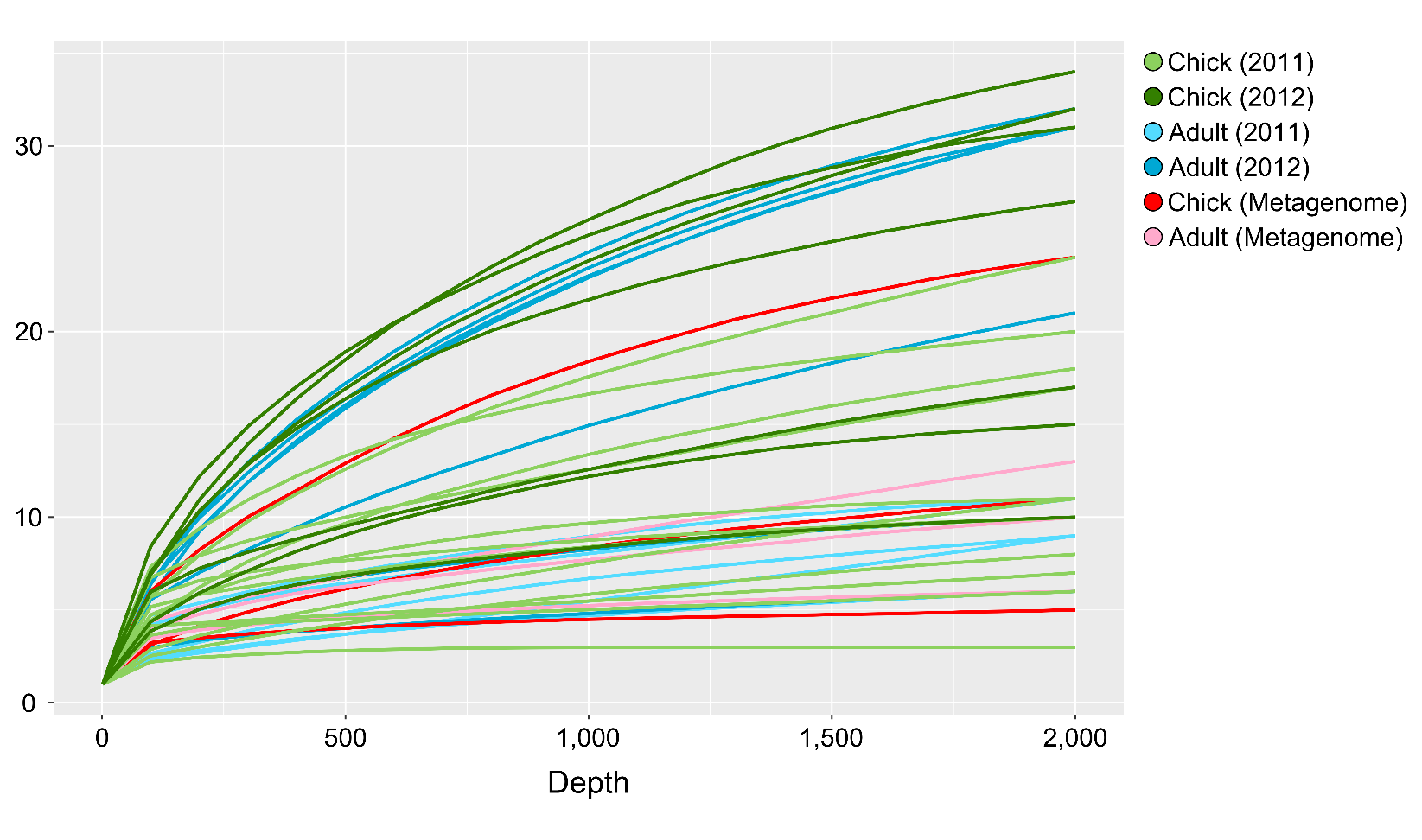
**

**Supplemental Figure S3. Rarefaction analysis of subsampled OTU table**


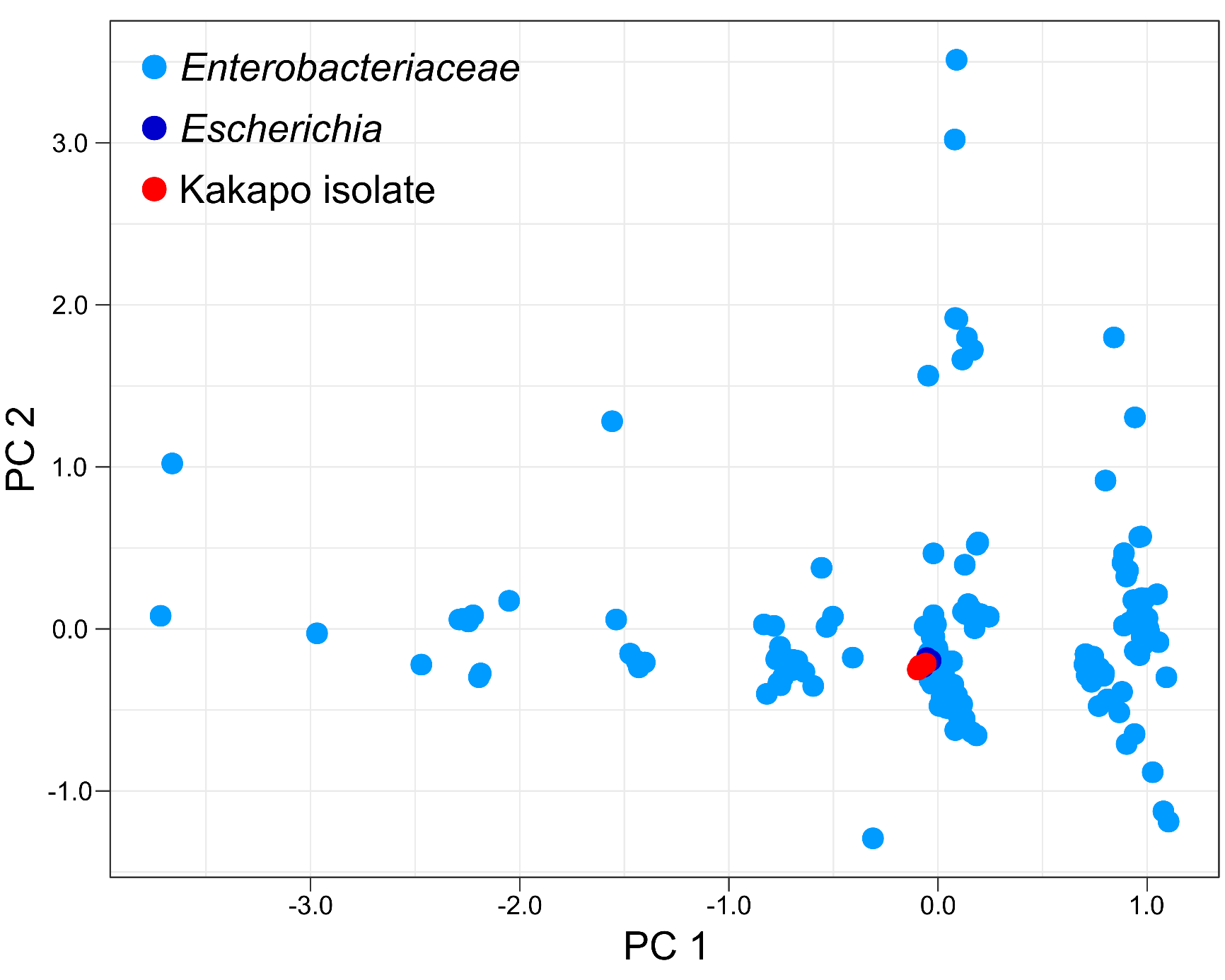


**Supplemental Figure S4. PCA ordination of KEGG-annotated *Enterobacteriaceae* genomes**

**
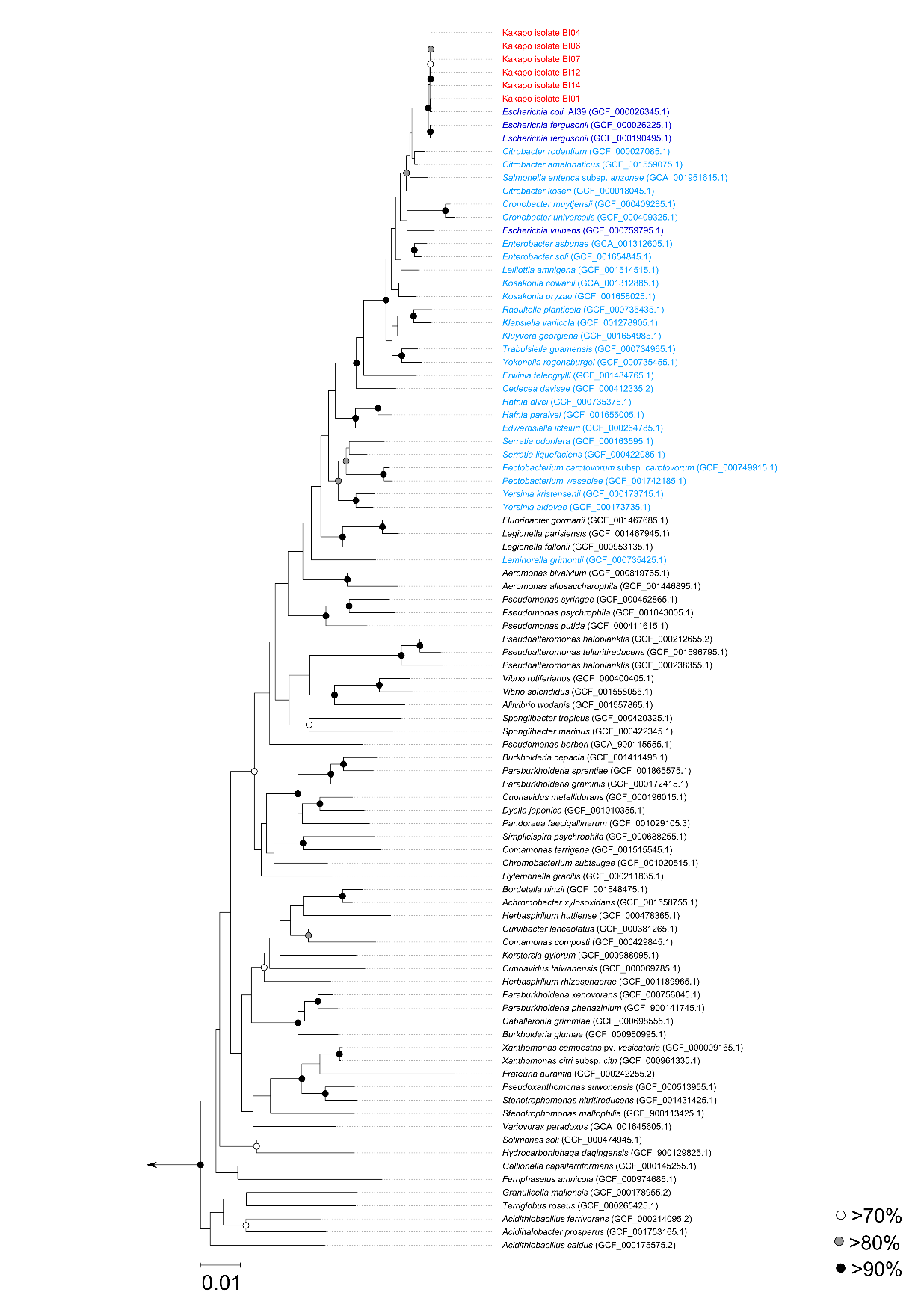
**

**Supplemental Figure S5. Phylogenetic inference of beta-1,4-endoglucan hydrolase sequences from Proteobacteria**

Kakapo isolate sequences are marked in red, with *Escherichia* in dark blue and other *Enterobacteriaceae* sequences in pale blue. Bootstrap support was determined through 100 resamplings.

**Table S1. Overview of the relationship between samples utilised for amplicon and metagenomic sequencing**

| Individual | Timepoint 1 | Metagenomic sampling | Timepoint 3 | Timepoint 4 |
| --- | --- | --- | --- | --- |
| Chick_1 |  |  |  | + |
| Chick_2 | + |  | + | + |
| Chick_3 | + |  | + |  |
| Chick_4 | + |  |  | + |
| Chick_5 | + |  |  | + |
| Chick_6 | + | + |  | + |
| Chick_7 | + | + |  | + |
| Chick_8 |  |  | + |  |
| Chick_9 | + | + | + | + |
| Chick_10 |  |  | + | + |
| Adult_1 | + | + |  | + |
| Adult_2 | + | + |  | + |
| Adult_3 | + |  |  | + |
| Adult_4 |  | + |  | + |
| Adult_5 | + |  |  | + |
| Adult_6 | + |  |  | + |
| Adult_7 | + |  |  | + |
| Adult_8 | + |  |  |  |
| Adult_9 | + |  |  |  |
| Adult_10 | + |  |  |  |

Columns labelled as Timepoint 1, 3 and 4 refer to sampling periods during the study design in (Waite *et al.*, 2014), following the exclusion of the hand-rearing phase (Timepoint 2 in original study). Additional sampling was performed for six individuals from which amplicon and metagenomic samples were generated.

**Table S2. Bacterial isolates used for genome sequencing**

| SampleID | Isolation conditions | Banding Pattern | Closest BLAST Match |
| --- | --- | --- | --- |
| BI01 | LB (aerobic) | 1 | *Escherichia fergusonii* |
| BI02 |  | 2 | *Escherichia fergusonii* |
| BI03 |  | 3 | *Escherichia coli* |
| BI04 | BHI (aerobic) | 2 | *Escherichia fergusonii* |
| BI05 |  | 3 | *Escherichia fergusonii* |
| BI06 | LB (anaerobic) | 2 | *Escherichia fergusonii* |
| BI07 |  | 3 | *Escherichia fergusonii* |
| BI08 | BHI (anaerobic) | 2 | *Escherichia fergusonii* |
| BI09 |  | 3 | *Escherichia fergusonii* |
| BI10 | M17 (anaerobic) | 2 | *Escherichia fergusonii* |
| BI11 |  | 3 | *Escherichia fergusonii* |
| BI12 | MRS (anaerobic) | 2 | *Escherichia fergusonii* |
| BI13 |  | 3 | *Escherichia fergusonii* |
| BI14 |  | 4 | *Streptococcus gallolyticus* |
| BI15 |  | 5 | *Streptococcus gallolyticus* |

List of bacterial isolates previously obtained from a previous cultivation experiment (Waite et al., 2013). Table layout is modified from original publication and highlighted isolates were used for genome sequencing in this study. The kakapo from which these isolates were obtained was also included in the metagenomic cohort (**Table S1**, Adult_4).

**Table S3. Quality control and assembly statistics for genome and metagenome samples**

| Sample | Raw reads (paired) | High quality reads (paired) | High quality reads (orphaned) | Assembled bases (MB) | Contigs | N50 | Predicted genes |
| --- | --- | --- | --- | --- | --- | --- | --- |
| BI01 (*E. fergusonii*) | 1,506,091 | 1,434,660 | 44,228 | 4.75 | 67 | 183,225 | 4,493 |
| BI02 (*E. fergusonii*) | 2,231,990 | 2,044,394 | 126,152 | 4.73 | 71 | 435,364 | 4,408 |
| BI04 (*E. fergusonii*) | 1,447,296 | 1,413,427 | 32,210 | 4.70 | 52 | 362,224 | 4,383 |
| BI06 (*E. fergusonii*) | 1,691,620 | 1,661,742 | 24,717 | 4.75 | 67 | 275,438 | 4,458 |
| BI07 (*E. fergusonii*) | 2,055,721 | 1,986,430 | 35,034 | 5.23 | 93 | 201,913 | 5,117 |
| BI12 (*E. fergusonii*) | 1,442,568 | 1,363,285 | 67,635 | 5.43 | 379 | 66,601 | 5,599 |
| BI14 (*S. gallolyticus*) | 1,725,202 | 1,667,360 | 54,060 | 2.18 | 121 | 34,542 | 2,236 |
| BI15 (*S. gallolyticus*) | 3,148,699 | 2,963,285 | 143,425 | 2.16 | 133 | 33,976 | 2,217 |
| Chick_6 | 3,236,242 | 3,096,959 | 135,783 | - | - | - | - |
| Chick_7 | 2,741,807 | 2,706,660 | 34,488 | - | - | - | - |
| Chick_9 | 2,890,590 | 2,857,683 | 31,822 | - | - | - | - |
| Adult_1 | 3,493,113 | 3,415,822 | 74,202 | - | - | - | - |
| Adult_2 | 2,334,971 | 2,288,253 | 45,279 | - | - | - | - |
| Adult_4 | 3,574,059 | 3,512,475 | 60,786 | - | - | - | - |
| Metagenomic assembly | 18,270,782 | 17,877,852 | 382,360 | 17.4 | 6,869 | 7,922 | 257,679 |

Reads used for metagenomics assembly (raw and quality filtered) are the sum of individual metagenomics samples.
